# An In-House ELISA for Treponema Antibodies in Bulk Milk as Part of a Monitoring Tool for Claw Health in Dairy Herds

**DOI:** 10.3390/vetsci10090571

**Published:** 2023-09-13

**Authors:** Menno Holzhauer, Jet Mars, Manon Holstege, Harold van der Heijden

**Affiliations:** 1 Bovine Health Department, Royal GD Animal Health, P.O. Box 9 7400 AA Deventer, The Netherlands; 2 R&D Department, Laboratory, Royal GD Animal Health, P.O. Box 9 7400 AA Deventer, The Netherlands; j.mars@gdanimalhealth.com (J.M.); h.vd.heijden@gdanimalhealth.com (H.v.d.H.); 3 Epidemiology and Statistics Department, Royal GD Animal Health, P.O. Box 9 7400 AA Deventer, The Netherlands; m.holstege@gdanimalhealth.com

**Keywords:** EAVLD, diagnostics, veterinary microbiology, monitoring and surveillance

## Abstract

**Simple Summary:**

Digital dermatitis (DD) is a painful infection of the skin at the coronary band of the claws, a major cause of lameness in dairy cattle and associated with several *Treponema* species (spp.). Clinical inspection of the feet is the common way to diagnose the disease, but is laborious. There are no simple diagnostic tools, which makes it difficult to monitor DD prevalence in a herd. Therefore, we developed, validated and implemented a Treponema antibody detecting ELISA for use in bulk tank milk. A weak relation between clinical scores of claws with the results of ELISA in individual milk samples was found. When using bulk milk, a clear increase in the ELISA response was observed when mean clinical scores of the claws in the herd were higher. Using the results of the Treponema ELISA in bulk milk, herds with a low or high number of cattle with DD could be distinguished. This ELISA is now being used in a claw health monitoring program for dairy cattle in the Netherlands. This helps farmers to obtain insight into the level of DD in their herd and is helpful to improve claw health in dairy cattle.

**Abstract:**

Digital dermatitis (DD) is a painful inflammation at the coronary band of the claws, a major cause of lameness in cattle and associated with infections with several *Treponema* spp. Clinical inspection of the feet is the best way to diagnose DD, but this is laborious and stressful for cattle. A simple diagnostic tool was developed to monitor DD prevalence at the herd level. An antibody ELISA based on antigens from four different *Treponema* spp. has been developed and validated in two field studies. In one study, bulk milk and individual milk samples of seven dairy herds, of which clinical claw scores were obtained, were tested. In the second study, bulk milk was tested from 110 herds of which clinical scores were obtained. A weak correlation between clinical scores of cows and the ELISA results in individual milk samples was observed. The ELISA response in bulk milk was higher in herds with higher mean clinical scores. Using the ELISA results in bulk milk, herds with a low or high proportion of cattle with DD lesions could be distinguished. This ELISA is useful to obtain insight into the DD status at the herd level, and is nowadays being used in a claw health monitoring program for dairy cattle in the Netherlands.

## 1. Introduction

Bovine digital dermatitis (DD) is a painful, infectious claw disorder that causes ulcerative lesions mainly at the coronary band of the hind legs of dairy cows and lameness [1,2]. The costs per case of DD were previous estimated at USD 132.96, for milk loss, decreased fertility and treatment costs [3,4]. This illustrates that DD is not only a serious issue in terms of animal welfare, but also has significant economic consequences [3,5,6]. Estimates for the prevalence of DD in the Netherlands range from 20% to 30% [7,8,9]. DD was first described in 1974, in Italy [10]. The most common bacteria associated with DD are multiple phylotypes from the genus *Treponema* [11]. Although the generally accepted opinion is that the local DD lesions are caused by a mixture of bacteria, the most representatives are *T. medium*/*T. vincentii*-like, *T. phagedenis*-like, and *T. pedis* bacteria according to the 16S rDNA and flaB2 gene homology [12,13,14,15,16]. 

Other bacteria found were *Dichelobacter nodosus* and *Fusobacterium necrophorum* [8] (Wilson-Welder, unpublished). The scoring system used in most studies and by claw trimmers is the M-score or a derivative thereof, according to a standardized classification for DD lesions, as developed by Döpfer et al. [17] and more extensively described by Berry et al. [18]. This is most often performed in the trimming chute or in the milking parlor [19,20] but is labor intensive. Current laboratory tests for DD diagnostics in individual cows are based on histology [1], cultivation [6], PCR techniques [21,22] and also serum ELISA of individual cows and bulk milk [23,24,25,26]. The advantage of the use of ELISA in milk is the more or less automatic availability of milk and the price of the ELISA in relation to histology and PCR. 

Reliable DD scoring enables macroscopic evaluation of DD lesions and is internationally recognized as the most accurate and detailed DD identification system [27]. Lifting the cow’s foot for inspection in the trimming chute continues to be the gold standard for DD detection; however, this is expensive, time consuming, labor intensive, and stressful for cattle [28,29,30]. Due to possible variations in the interpretation of visual lesions, the system might lead to misclassification. Standardized trimming chute inspections are only practical for assessing disease prevalence for special epidemiological studies and topical treatment or good evaluation of treatment. Regular claw trimming in West-European countries is only performed twice or a maximum of three times per lactation. In the meantime, it is useful for dairy farmers to obtain information about the infection pressure of *Treponema* spp. in the dairy herd. 

The objective of this study was to develop, validate and implement a Treponema an-tibody ELISA in bulk milk to monitor and assess DD prevalence at the herd level.

## 2. Materials and Methods

### 2.1. Farms in This Study

The farms used in this study were chosen by convenience sampling based on the willingness of the farmers to participate, geographical location and DD prevalence (<10%, 10–25% and >25%) last estimated by the claw trimmer at the moment of regular preventive trimming. We attempted to create an extensive database of dairy cattle that includes herds with varying DD prevalence in order to include enough variability and power for the analysis. The database was based on a total of 110 farms, visited between August 2017 and July 2018, and young animals (<21 months) were excluded. Seven herds, determined by the claw trimmer to have few, moderate and severe DD, were visited by a GD employee (trained by MH before the start of this study), three times, with 6 months between each visit, at the moment of regular preventive claw trimming. Inter-observer agreement for the M-score between the observers was tested with photographs by using Cohen’s kappa coefficient (k) (interpretation of kappa: fair if 0.21 ≤ k ≤ 0.40, moderate if 0.41 ≤ k ≤ 0.60 and substantial if 0.61 ≤ k ≤ 0.80) [31]. All observers scored 42 photographs of hind claws for DD.

### 2.2. Herd Data Collection

In seven herds, at that moment of regular claw trimming, all claws were checked 3× with 6 month intervals by trained employees, scored and noted according to the M-score system for the presence and severity of DD [17,18], and claws were trimmed and treated topically by the claw trimmer when necessary. Four weeks after the each trimming, all lactating cows were milk sampled individually at the moment of milking. This data collection in the 7 herds took place between August 2017 and August 2018. For the second part, of 110 herds, data from one regular claw trimming, DD scores were noted by trained employees and a bulk milk sample was taken. These herds were visited between August 2017 and January 2018. The score system used in this study was applied according a standardized classification for DD lesions, as developed by Döpfer et al. [17] and more extensively described by Berry et al. [18]. This classification comprises six distinct classes (M0, M1, M2, M3, M4, and M4.1). Class M0 is described as skin where lesions are macroscopically absent, class M1 as an active granulomatous area of 0–2 cm, class M2 as an ulcerative lesion of >2 cm, class M3 as an ulcerative lesion covered by a scab, class M4 as alteration of the skin with hyperkeratotic lesions that can have a proliferative aspect, and class M4.1 was scored for the same scar tissue as for M4, but with a new small lesion, as seen in the M1 stage [8,17,18]. Studies on DD tend to focus on these lesions because they can cause lameness and are considered to be infectious [32]. Before the first moment of DD scoring of dairy cows, 7 students were trained by the first author at the moment of a regular trimming and agreement (Cohen’s kappa coefficient) was tested on 42 photographs of DD lesions before the official scoring.

### 2.3. Samples

The dataset for laboratorial investigation comprised individual milk samples of lactating dairy cows of 7 herds, collected 3 times, 4 weeks after previous trimmings (*n* = 897 cows). In herds (*n* = 110), from which we used data (M-scores) of one regular claw trimming, bulk milk samples on the day of claw trimming and for one year, up to 5 additional bulk milk samples from these herds were collected with approximately 10 week intervals.

### 2.4. Laboratorial Analysis of the Milk Samples

#### Treponema Antigen Preparation

The milk samples were tested for antibodies against *Treponema* spp. using an indirect ELISA based on whole-cell antigens [23]. Briefly, *Treponema* spp. were cultured, harvested by centrifugation, washed in PBS twice and disrupted by ultrasound treatment. The suspension was centrifuged at 5000× *g* to remove insoluble particles, and the supernatant was aliquoted and stored at −20 °C until used.

High-binding microtiter plates (Greiner Bio-one BV, Alphen aan den Rijn, The Netherlands) were coated with a mixture of *T. medium/T. vincentii*-like, *T. phagedenis*-like, and two strains of *T. pedis* bacteria of which one was formerly known as *T. denticola* (100 µL per well) in coating buffer and incubated overnight at 4 °C. Then, 100 µL/well blocking solution was added and plates were incubated for 1 h at room temperature. Subsequently, the contents of the plates were discarded, and plates were dried for four hours at 37 °C, vacuum sealed and stored at 4–8 °C. Plates were incubated with 100 µL per well of either sera diluted 1:100 diluted in assay buffer, non-diluted individual milk samples or non-diluted bulk milk samples for 1 h at 37 °C after washing five times with washing buffer (PBS + 0.05% Tween 20) using a Biotek automatic washing station. Then, 100 µL per well diluted horseradish peroxidase (HRP)-conjugated Protein G (Abcam, Cambridge, UK) in assay buffer was added and the plates were incubated for 1 h at 37 °C. Subsequently, the plates were washed again. Then, 100 µL/well TMB (IDEXX Laboratories, Westbrook, NJ, USA) was added, and the plates were incubated for 30 min at room temperature. The reaction was stopped by adding 50 µL/well 0.5 M sulfuric acid (VWR International BV, Amsterdam, The Netherlands). The optical densities (OD) were measured at 450 nm using an ELISA microplate reader (BioTek Instruments, Inc., Winooski, VT, USA). S/P values were calculated by the formula: S/P = (OD Sample–OD Negative control)/(OD Positive control–OD Negative control).

### 2.5. Statistical Analysis

Using Stata version 17.0 [33], tables and figures, i.e., boxplots and scatterplots, were generated to describe the data. The results of the agreement (Cohen’s kappa coefficient) for the M-score of the students who performed the M-scores in the herds were determined. Using a linear regression model (significance limit at *p* < 0.05) only including 1 covariate (X), assuming a linear association, the coefficient of determination (r^2^) was determined between X and Y. X was either the individual or the bulk milk S/P ratio and Y could be the individual S/P ratio; the M2-score was the prevalence or the average M-score depending on the association that was investigated.

## 3. Results

The mean number of cows trimmed in the first visits to the seven herds that were scored 3 times was 98 (SD: 14.1) and 63% of these cows had an M-score ≠ 0. The percentage of cows with an active lesion (M2) in the 110 herds varied from 2% to49% at the herd level. The number of records collected per farm varied between 94 and 262 (mean = 174,6; SD = 42.8). From these records, 38.5% of animals were diagnosed with one of the DD stages other than M0 in one or both hind legs. The leg prevalence for each of the DD stages is shown in Table 1.

The results of the agreement (Cohen’s kappa coefficient) for the M-score of the students who performed the M-scores in the herds varied between 0.44 and 0.71. Six out of seven students were in the category ‘moderate’ and one was in the category ‘substantial’. The overall kappa value was 0.51 (moderate).

**Figure 1:**
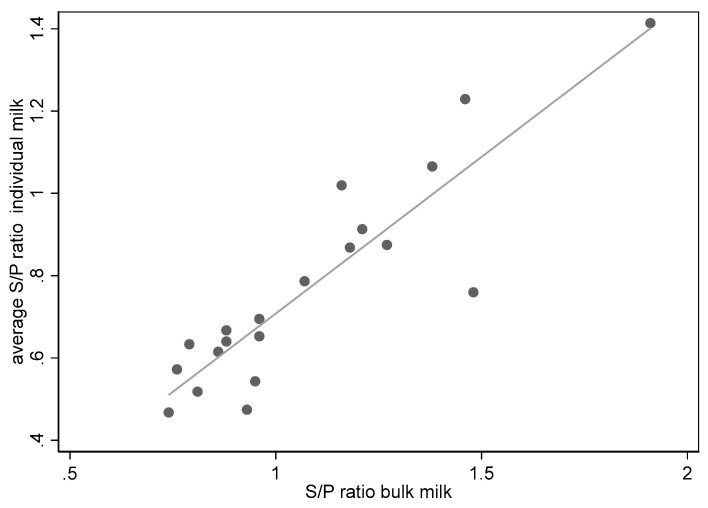
Association of bulk milk samples and average ELISA S/P ratios of individual milk samples on seven dairy herds during three visits (*n* = 20, one bulk sample of one herd was not available).

The milk samples of all individual cows and the bulk milk samples of the seven herds obtained during three visits and tested in the ELISA showed a good association (*n* = 20, *r*^2^ = 0.82, *p* < 0.001) between the average S/P ratio of the individual samples and the S/P ratio of the bulk milk samples (see also Figure 1).

**Figure 2:**
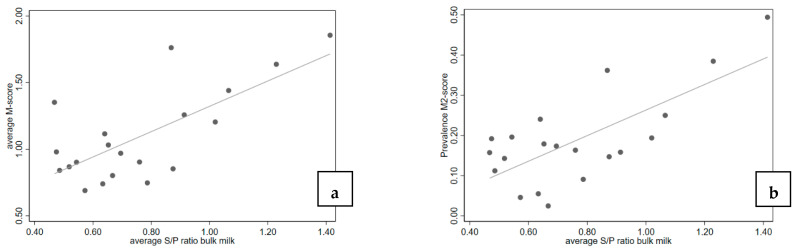
Scatterplot of trimming scores and bulk milk ELISA S/P ratios on seven herds sampled thrice; (**a**) average M-score and S/P ratio (*n* = 20, *r*^2^ = 0.504, P); (**b**) M2 prevalence and S/P ratio (*n* = 20, *r*^2^ = 0.513, *p* < 0.001).

The ELISA results in individual milk samples in these seven herds showed a slight increase in S/P ratio related to the age of cows. The mean S/P ratio was 0.5 in cows aged 2 years and increased to the mean S/P ratio 0.9 in cows 6 years of age. When the average S/P ratios of the individual milk samples in ELISA were compared against the average M-scores or the M2 prevalence in these herds, in both cases a weak, but statistically significant association between the severity of the lesions and the S/P ratio was found (see also Figure 2).

**Figure 3:**
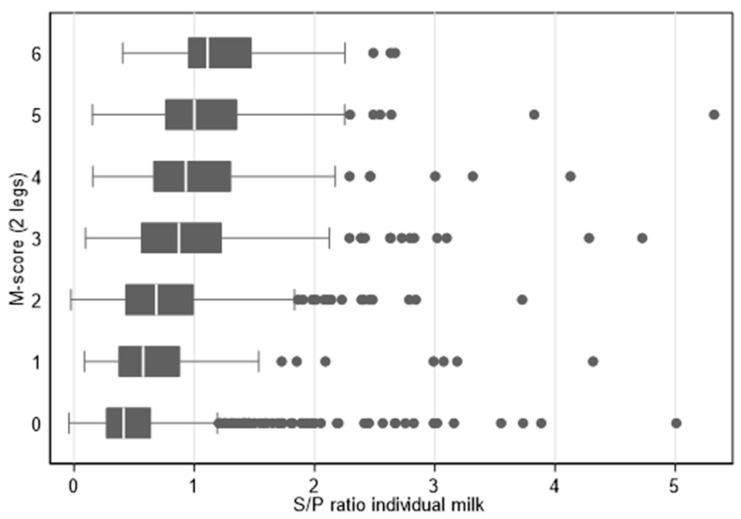
Boxplots of individual milk ELISA S/P ratios for cows with different total M-scores on seven herds during three samplings (*n* = 1667 cow-milking combinations).

An alternative total M-score was calculated for each animal depending on the severity of the lesions. The (standard) M-score 2 counted as 3, an M1, M3, or M4.1 score as 2, an M4-score as 1, and an M0-score as 0. The total M-score of a cow was the total score of both hind legs (i.e., a total M-score of 6 means that both hind legs had an M2-lesion; a total M-score of 0 means an M0 score for both hind legs). There was a general increase in the ELISA S/P ratio with the higher alternative total M-score (see also Figure 3).

To distinguish between herds with high and low levels of antibodies against *Treponema* spp. in the bulk milk, two cut-off values were determined based on the 25 percentile and 75 percentile of the S/P ratio obtained for all the bulk milk samples from the seven herds. The lower cut-off was 0.87, while the higher cut-off was 1.24.

**Figure 4:**
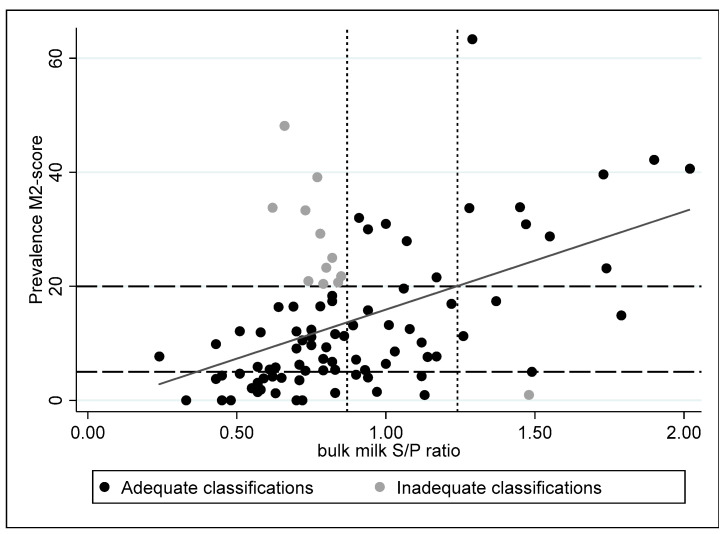
Comparison of M-scores and bulk milk ELISA S/P ratios on 95 herds with information on both M2-scores and the bulk milk S/P ratio (*r*^2^ = 0.23, *p* < 0.001); the dotted (S/P ratio) and interrupted line (ELISA) represent the set cut-offs.

These ELISA cut-offs were evaluated in the 110 herds of the second part of this study. Herds with a prevalence of M2 lesions higher than 20% were considered to have a high rate of clinical lesions, while herds with a prevalence of M2 lesions lower than 5% were considered to have no or few lesions. The herds with an M2 prevalence between 5% and 20% were herds with intermediate clinical signs. One of the herds had a high ELISA S/P ratio in bulk milk, but a low M2 prevalence, while 11 herds were found with a high M2 prevalence and a low ELISA S/P ratio (Figure 4).

### Treponema ELISA Bulk Milk Program

**Figure 5:**
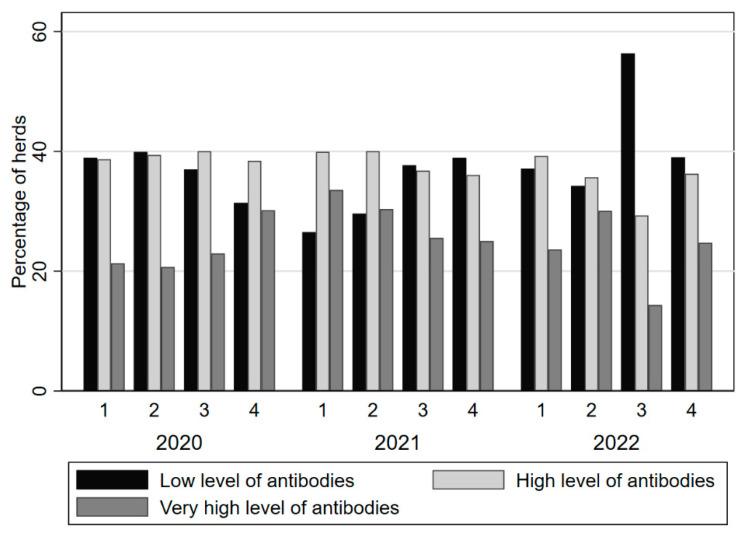
Quarterly percentage of farms in a specific *Treponema* bulk milk antibody category in the period 2020–2023.

From 2019, the Treponema ELISA was added to the Royal GD claw health program, using quarterly bulk milk testing. Since 2020, the S/P ratios are reported and the result is categorized into ‘little or no antibodies’, ‘many antibodies’ or ‘very many antibodies’. In 2022, 1558 herds participated in the Treponema bulk milk program. Per quarter, over the period 2020–2022, the percentage of farms with ‘little or no antibodies’ was 37.2% (minimum 26.5 and maximum 56.3%; Figure 5). The average quarterly percentage of herds with many antibodies was 37.5% (minimum 29.3% and maximum 40.0%) and the average quarterly percentage of herds with very many antibodies was 25.2% (minimum 14.4% and maximum 33.6%; see also Figure 5).

## 4. Discussion

The purpose of the present study was to develop and evaluate an ELISA for detection of digital dermatitis (DD)-related antibodies against *Treponema* spp. in bulk tank milk (BTM). Promising results were found when the ELISA S/P ratios were compared with the number of legs affected and the severity of DD in those herds.

Our indirect ELISA used a mixture of crude antigens from *Treponema* spp. related to DD [12,13]. In the first phase of this study, the ELISA was evaluated in seven herds, where all cows were sampled individually 4 weeks after the moment individual cows were inspected and scored for DD lesions during three visits. Additionally, BTM samples were obtained from these herds at the same moment of individual sampling. The ELISA results of the BTM samples showed good agreement with results from individual cows (*r*^2^= 0.82), which was also observed in another study [22]. Additionally, in general, a fairly good relation (*r*^2^ = 0.51) was found between the ELISA results and the severity of the lesions in the individual animals, a good correlation between the clinical presentation of the different M-stages on the hind legs and the BTM ELISA S/P ratios. However, the ELISA is not intended for use as an individual diagnostic tool. Additionally, a good correlation was seen between the average M-score or the prevalence of the M2-lesion and the BTM ELISA S/P ratios in these herds (*r*^2^ = 0.23). During the clinical observations, no attention was paid to other disorders such as udder cleft dermatitis, teat necrosis and hock lesions. This type of lesions have also been associated with the presence of *Treponema* spp., might have influenced the ELISA results [34,35], and could have led to a reaction in the ELISA in the absence of DD lesions.

Our findings in individual animals are in line with the results of Gomez et al. [23], although blood serum samples from heifers were tested in that study. It was concluded that heifers never diagnosed with DD lesions kept constant low levels of Treponema antibodies, indicating that although detectable levels of immune response can be observed in endemically infected herds, lack of clinical disease is consistent with a significantly lower and constant response over time [1]. Furthermore, a dramatic increase in antibodies was observed in heifers upon diagnosis of an M2 stage, reflecting a clear implication of *Treponema* spp. in the pathogenesis of clinical DD, as extensively reported earlier [6,36,37].

In a study in Sweden, an ELISA for detection of antibodies against *T. phagedenis* was performed to evaluate the discrimination between infected and non-infected cows and a BTM test was used to quantitatively evaluate the level of antibodies in BTM and the sum of serological responses of the individual cows [24]. In a French study, a *T. phagedenis* antibody ELISA was applied on BTM samples to accurately estimate the DD within-herd prevalence. They concluded that the BTM antibody ELISA showed great promise at the herd level for screening purposes during DD management programs [25]. Finally, in a study from Liverpool University, an indirect ELISA was assessed to estimate the suitability for the diagnosis and severity assessment of DD in cattle. They concluded that although the individual levels of anti-Treponema antibodies were associated with the presence of DD, the ELISA failed to detect disease unequivocally and had no predictive value in the future occurrence of DD lesions [38].

The results of the first phase of our study were used to determine cut-offs for the ELISA using BTM samples. That was based on the P25 percentile and the P75 percentile of the BTM ELISA results, respectively. In that way, BTM samples with high levels of antibodies (>P75) could be differentiated significantly from BTM samples with low levels of antibodies (<P25). The ELISA cut-offs for BTM samples were validated in the second phase of the present study, where BTM samples were taken in 110 herds four weeks after trimming, upon which M-scores of all animals were also registered. It was found that herds with high levels of BTM antibodies (>P75) in general were also the herds with a high prevalence (>20%) of M2 lesions, while herds with a low level of BTM antibodies (<P25) had less than 10% M2 lesions. The observed variation might be caused by the age (parity) of the animals in the herd, different *Treponema* spp. in the areas where the studies were performed, infection pressure, single or repeated infection, management and housing. The observed variation might be partly associated with the age of the animals, due to the higher S/P ratio observed in older cows that probably have had repeated infections. Other sources of variation might be due to infections with other *Treponema* spp., change in infection pressure per herd or in time, management and housing. In older studies, cross-reacting antibodies to *Treponema pallidum* and *Treponema phagedenis* due to *Borrelia burgdorferii* infections have been described in humans [39,40]. It is unknown whether Borrelia infections occur in cattle in the Netherlands, although recently in Belgium, a seroprevalence of 12.9% was reported [40]. A serological assay was not available, therefore we could not explore this issue further. 

Another factor causing discrepancy between the bulk milk results and the DD observation might be correlated to other lesions in cattle related to Treponema infections, such as non-healing claw disorders, udder cleft dermatitis and skin lesions [35,41,42]. 

As in the study of Gomez et al. [23], in our study, the presence of the M2 stages was correlated with the highest serological response. So far, we have not examined the influence of treatment on the antibodies in BTM. It would be interesting to study ELISA reactions after treatment of DD. It would be helpful as a tool for farmers when BTM ELISA S/P ratios would drop after successful treatment of DD. In a Norwegian study, bulk milk samples were collected from 154 dairy herds with high and low DD prevalence. That study showed that ELISA tests could detect antibodies against DD-associated Treponema spp. in bulk tank milk from Norwegian dairy herds. However, due to the mild lesions and low within-herd prevalence, different ELISA tests could not produce an acceptable sensitivity for detection of DD. However, the ELISA tests of bulk tank milk was considered a useful supplement [26]. 

In most countries, regularly claw trimming is performed two or three times per lactation. Where especially chronic DD stages do not result in lameness, but are infectious for susceptible animals [43], it is for dairy farmers to obtain useful information about the infection pressure of DD causing *Treponema* spp. in the dairy herd. Laboratory support for individual diagnosis is less useful where, at claw trimming, the clinical presentation of different M-stages is so obvious that this is sufficient to start treatment.

We conclude that the Treponema antibody ELISA is useful for monitoring on the herd level, but not for individual diagnosis.

In the Netherlands, the number of dairy herds that took part in our Treponema monitoring program steadily increased to 1630 in May 2023.

To summarize, periodically, BTM testing for antibodies against *Treponema* spp. is valuable to monitor the DD prevalence at the herd level and may support dairy farmers in applying the correct curative and preventive measures [20].

## 5. Conclusions

A Treponema bulk milk ELISA was developed to obtain insight into the DD status at the herd level, and the ELISA is suitable to be used in a claw health monitoring program for dairy cattle in the Netherlands. 

## Figures and Tables

**Table 1:** Leg-level prevalence of all M-stages of digital dermatitis in hind legs of Dutch dairy cows (*n* = 110 herds and *n* = 21,560 hind legs).

M-Stage	Prevalence	Frequency
M0	61.4%	13,246
M1	1.4%	295
M2	7.1%	1534
M3	0.3%	75
M4	20.9%	4512
M4.1	8.8%	1891

## Data Availability

All data are available from the authors for up to 5 years after publication.
